# The destruction complex of beta-catenin in colorectal carcinoma and colonic adenoma

**DOI:** 10.1590/S1679-45082016AO3678

**Published:** 2016

**Authors:** Guilherme Muniz Bourroul, Hélio José Fragoso, José Walter Feitosa Gomes, Vivian Sati Oba Bourroul, Celina Tizuko Fujiyama Oshima, Thiago Simão Gomes, Gabriela Tognini Saba, Rogério Tadeu Palma, Jaques Waisberg

**Affiliations:** 1 Hospital do Servidor Público Estadual “Francisco Morato de Oliveira”, São Paulo, SP, Brazil.; 2 Escola Paulista de Medicina, Universidade Federal de São Paulo, São Paulo, SP, Brazil.; 3 Faculdade de Medicina do ABC, Santo André, SP, Brazil.

**Keywords:** Colorectal neoplasms, Adenoma, Immunohistochemistry, beta Catenin, Genes, APC, Glycogen synthase, Axin protein, Ubiquitin, Wnt signaling pathway

## Abstract

**Objective:**

To evaluate the destruction complex of beta-catenin by the expression of the proteins beta-catetenin, adenomatous polyposis coli, GSK3β, axin and ubiquitin in colorectal carcinoma and colonic adenoma.

**Methods:**

Tissue samples from 64 patients with colorectal carcinoma and 53 patients with colonic adenoma were analyzed. Tissue microarray blocks and slides were prepared and subjected to immunohistochemistry with polyclonal antibodies in carcinoma, adjacent non-neoplastic mucosa, and adenoma tissues. The immunoreactivity was evaluated by the percentage of positive stained cells and by the intensity assessed through of the stained grade of proteins in the cytoplasm and nucleus of cells. In the statistical analysis, the Spearman correlation coefficient, Student’s *t*, χ^2^, Mann-Whitney, and McNemar tests, and univariate logistic regression analysis were used.

**Results:**

In colorectal carcinoma, the expressions of beta-catenin and adenomatous polyposis coli proteins were significantly higher than in colonic adenomas (p<0.001 and p<0.0001, respectively). The immunoreactivity of GSK3β, axin 1 and ubiquitin proteins was significantly higher (p=0.03, p=0.039 and p=0.03, respectively) in colorectal carcinoma than in the colonic adenoma and adjacent non-neoplastic mucosa. The immunohistochemistry staining of these proteins did not show significant differences with the clinical and pathological characteristics of colorectal cancer and colonic adenoma.

**Conclusions:**

These results suggest that, in adenomas, the lower expression of the beta-catenin, axin 1 and GSK3β proteins indicated that the destruction complex of beta-catenin was maintained, while in colorectal carcinoma, the increased expression of beta-catenin, GSK3β, axin 1, and ubiquitin proteins indicated that the destruction complex of beta-catenin was disrupted.

## RESUMO

**Objetivo:**

Avaliar o complexo de destruição da betacatenina no carcinoma colorretal e no adenoma do colo pela expressão das proteínas betacatenina, *adenomatous polyposis coli*, GSK3β, axina e ubiquitina.

**Métodos:**

Amostras de tecidos de 64 doentes com carcinoma colorretal e de 53 pacientes com adenoma do colo foram analisadas. Blocos de tecidos foram submetidos ao estudo imuno-histoquímico com anticorpos policlonais nos tecidos do carcinoma, mucosa não neoplásica adjacente e adenoma. A imunorreatividade foi avaliada pela porcentagem de positividade de células coradas e pela intensidade do grau de coloração das proteínas no citoplasma e no núcleo das células. Na análise estatística, foram utilizados o coeficiente de correlação de Spearman, os testes *t* de *Student*, χ^2^, Mann-Whitney e de McNemar, e a análise de regressão logística univariada.

**Resultados:**

No carcinoma colorretal, as expressões da betacatenina e da *adenomatous polyposis coli* foram significativamente maiores do que em adenomas do colo (p<0,001 e p<0,0001, respectivamente). A imunorreatividade das proteínas GSK3β, axina 1 e ubiquitina foi significativamente maior (p=0,03, p=0,039 e p=0,03, respectivamente) no carcinoma colorretal do que no adenoma e na mucosa não neoplásica adjacente. A coloração imuno-histoquímica dessas proteínas não apresentou diferenças significantes em relação às características clinicopatológicas do câncer colorretal e do adenoma.

**Conclusões:**

Em adenomas, as menores expressões de betacatenina, axina 1 e GSK3β indicaram que o complexo de destruição da betacatenina estava conservado, enquanto que, no carcinoma colorretal, o aumento das expressões da betacatenina, GSK3β, 1 axina, e ubiquitina indicaram que o complexo de destruição de betacatenina estava alterado.

## INTRODUCTION

All cells exist under strict regulation of signals for growth, apoptosis, differentiation, cell-cell interactions and cell-extracellular matrix interactions.^(1)^ Colorectal cancer (CRC) is considered the result of the cumulative effect of multiple mutations within the cell that allow it to escape growth control and regulatory mechanisms.^(1,2)^ It has become apparent that the accumulation of gene mutations in a clonal cell results in the transition from the normal colon epithelial cell into colorectal carcinoma.^(3)^

Beta-catenin, a member of the catenin protein family, is a dual-function protein that regulates the coordination of cell-cell adhesion and gene transcription.^(4)^ Beta-catenin is a subunit of the cadherin protein complex and acts as an intracellular signal transducer in the Wnt signaling pathway.^(3,4)^ The canonical Wnt signaling pathway, acting through beta-catenin, modulates a variety of cellular processes, including proliferation, survival, apoptosis, differentiation, cell adhesion and motility.

The Wnt effector beta-catenin is a transcriptional co-activator that can also mutate to a potent oncogene, while the canonical Wnt signaling pathway stabilizes beta-catenin transcription.^(4,5)^ Mutations and the overexpression of beta-catenin are associated with many cancers, including hepatocellular carcinoma, colorectal carcinoma, lung cancer, malignant breast tumors, and ovarian and endometrial carcinomas.^(6)^

**Figure 1:**
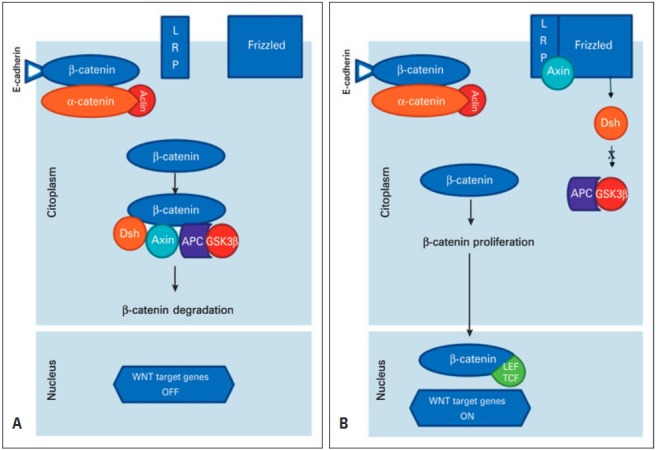
The Wnt/beta-catenin signaling pathway. (A) In the presence of the destroyer complex, beta-catenin is degraded via adenomatous polyposis coli/axin/glycogen synthase kinase 3β. (B) When the binding of beta-catenin with the complex by destroying not occurs, then the beta-catenin is not phosphorylated and this protein penetrates the cell nucleus. Inside the nucleus, the beta-catenin binds to the transcription factor target genes lymphoid enhancing factor/T-cell factor to promote carcinogenesis

LRP: lipoprotein receptor-related protein; Dsh: Dishevelled; APC: adenomatous polyposis coli; GSK3β: axin/glycogen synthase kinase 3β; LEF: lymphoid enhancing factor; TCF: T-cell factor.

Excess cytoplasmic beta-catenin is incorporated into multi-subunit complex which includes the destruction of proteins axin 1, adenomatous polyposis coli (APC), casein kinase 1 (CK1), and glycogen synthase kinase 3β (GSK3β).^(4)^ In the absence of a Wnt signal, beta-catenin binds to the APC tumor suppressor protein and this protein is recruited to the destruction complex, which promotes its phosphorylation by axin, CK1 and GSK3β.^(1,3,4,6)^ The phosphorylation of beta-catenin by GSK3β leads to its ubiquitination forming the complex polyubiquitin/beta-catenin and, subsequently, is degraded into the proteasome.^(4,6)^The mutation of genes correspond to the formation of the complex destruction of the beta-catenin protein and triggers a cascade of events that lead to the disruption of the APC/axin 1/GSK3β complex and accumulation of unphosphorylated beta-catenin in the cytoplasm.^(1,4)^ Then, the beta-catenin stabilized protein translocates into the nucleus, in which it activates the expression of a range of genes in association with the T-cell factor (TCF) and the lymphoid-enhancing factor (LEF), both transcription factors, that, in turn, activate the target genes that are related to carcinogenesis (Figure 1).^(1,3,4)^

The so-called adenoma-carcinoma sequence nowadays still represents the paradigm and the support of our understanding of the molecular and genetic basis of this disease. In human CRC, Wnt signaling activation is hypothesized to be the key event for adenoma initiation, whereas additional mutations are required for the progression from adenoma to carcinoma.^(2-4)^ A large body of evidence has suggested that aberrant activation of Wnt signaling following APC loss is a major cause of colon adenoma formation.^(4-6)^ Loss-of-function and gain-of-function studies of Wnt proteins and beta-catenin have suggested that aberrant Wnt signaling activation after APC loss is responsible for the initiation of intestinal adenoma.^(3,7)^ Therefore, hyperactivation of Wnt signaling is generally viewed as the key event for the initiation of intestinal adenoma after APC loss.^(4,8)^ An important step is to elucidate the molecular mechanisms underlying the cooperative functions of Wnt signaling, which may lead to the identification of novel therapeutic targets for the prevention and treatment of gastrointestinal cancer.^(6,8)^

The genetic mutations accumulated during tumor development are responsible for the deregulation of key signal transduction pathways, such as Wnt, which is responsible for uncontrolled cell growth, inhibition of apoptosis, and immortalization.^(2,4,5)^ Still, little is known about the multiple interactions and mutual influences of this defective pathway, and about the mechanisms responsible for the genetic instability contributing to the establishment of the multiple genetic defects that are necessary to promote tumor progression and malignancy.^(1,2,4)^

By defining the molecular alterations involved in the development of the sporadic CRC, it is possible to expect the achievement of specific molecular targeting for the treatment of already-established tumors, as well as for chemoprophylaxis interventions.^(7,8)^

## OBJECTIVE

To evaluate the destruction complex of beta-catenin by the expression of beta-catetenin, adenomatous polyposis coli, glycogen synthase kinase 3β, axin, and ubiquitin proteins in colorectal carcinoma and colonic adenoma. By defining this protein alterations of the Wnt signaling pathway involved in the adenoma and colorectal cancer, we can to expect the achievement of specific molecular targeting for the treatment of already established tumors.

## METHODS

This is an observational, longitudinal and retrospective study. A total of 64 patients with CRC and 53 patients with colonic adenoma were studied. Between 2006 and 2010, 64 patients with CRC were consecutively submitted to curative surgery with resection of their colorectal tumors. Patients with colonic adenoma underwent the removal of their adenomas by colonoscopy.

The inclusion criteria were adult patients with CRC or colorectal adenoma confirmed by histological analysis. Exclusion criteria were the presence of colorectal hereditary nonpolyposis neoplasm (Lynch syndrome), CRC associated with inflammatory bowel disease, and intestinal polyposis syndromes.

In patients submitted to surgery for CRC, 64 tissue samples were obtained from the tumor and from adjacent non-neoplastic colorectal mucosa, located 10cm from the upper margin of the neoplasm. In the 53 patients with colonic adenomas, there were 71 resected adenomas.

Among the patients operated with CRC, 33 (51.6%) were females. The median age was 69.2±7.4 years (51 to 90 years). The location of the CRC was in the colon in 38 (59.4%) patients and in the rectum in 26 (40.6%).

Regarding the adenomas, 27 (50.9%) patients were male. The median age was 60.7±3.4 years (29 to 88 years). The location of the adenomas was 46 (86.8%) in the left colon and 7 (13.2%) in the right colon. There were no rectal adenomas.

Within patients with CRC, information was recorded regarding the location, size, level of invasion in the intestinal wall, inflammatory infiltrate degree, lymph node involvement, degree of tumor differentiation, lymphatic-vascular-neural invasion, Classification of Malignant Tumours (TNM),^(9)^ presence of synchronous metastases, and immunostaining (staining intensity and percentage of stained cells scores) of the antibodies that were used in the colorectal tissue. In patients with colonic adenoma, statistics were recorded regarding the morphological characteristics of the neoplasm (location, histological type, and degree of cell atypia) and the immunostaining (staining intensity and percentage of stained cells scores) in the colonic tissue.

The tissues were fixed in formalin and routinely processed by using the paraffin-embedding method for histological analysis. Histological sections with 3µ thickness were obtained from each block. All slides were stained with hematoxylin-eosin (HE) and revised by the pathologist for confirmation of the diagnosis.

In the stained slides, the areas of the tumor were identified for the preparation of tissue microarray (TMA). The TMA block was prepared by using Beecher™ (Beecher Instruments, Silver Spring, MD, USA) equipment, in according to the previously described protocol that was used in our laboratory.^(10)^ The paraffin blocks were cut 3µm thick, and the slides were prepared for immunohistochemical study. The antibodies which were used included: rabbit polyclonal primary anti-beta-catenin; rabbit polyclonal anti-APC; rabbit polyclonal primary anti-GSK3β; rabbit polyclonal primary anti-axin; mouse polyclonal primary anti-ubiquitin (all from Santa Cruz Biotechnology, Inc., Santa Cruz, CA, USA). All antibodies were used at a dilution of 1:100. The positive controls used included normal colon tissue for APC and axin, HeLa whole-cell lysate for GSK3β and ubiquitin, and human colon cancer for beta-catenin. A similar slide was used as a negative control, subtracting the primary antibody from the reaction.

**Figure 2:**
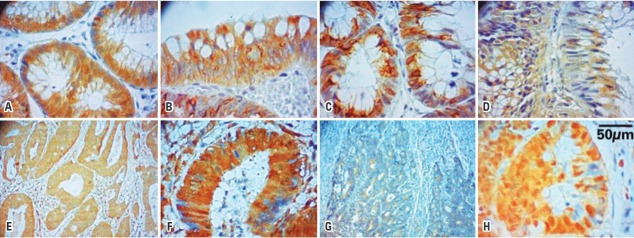
Photomicrographs of the immunohistochemical staining of the proteins represented by the brownish color in the cytoplasm of cells. Beta-catenin (A – colonic adenoma, 200X; B – colorectal carcinoma, 200X); adenomatous polyposis coli (C – colonic adenoma, 200X; D – colorectal carcinoma, 200X); GSK3β (E – colonic adenoma, 100X; F – colorectal carcinoma, 200X); axin 1 (G – colorectal carcinoma, 100X); ubiquitin (H – colorectal carcinoma, 200X)

The anti-beta-catenin, anti-APC and anti-GSK3β antibodies were used in CRC tissue, colonic adenoma and in adjacent non-neoplastic mucosa. The anti-axin and anti-ubiquitin antibodies were used in CRC tissue and adjacent non-neoplastic mucosa (Figure 2).

The immunoexpression score was prepared according to the method described by Hao et al.^(6)^ Positivity was considered to be a score zero if less than 5% of epithelial cells were stained in the neoplasm; a score 1 if there was 5% to 25%; a score 2 if there was 26% to 50%; a score 3, if there was 51% to 75%; and a score 4, if more than 75% of epithelial cells were stained in the neoplasm. The intensity was considered to be score zero when there were no stained cells; a score 1 when the stain was weak; a score 2 when the stain was moderate; and a score 3 when the intensity of stained cells was strong. The final score of immunoexpression, which varied between zero and 12, was obtained by multiplying the scores of intensity and positivity. The immunoexpression was classified as reduced when the final score was among zero and 8, and strong if the final score was among 9 and 12.

All slides were analyzed by two well-trained, independent researchers who had no access to any pathological information. The final score (zero to 12) was the result of a mean score by the researches. In most cases, there was agreement between the two evaluations of the final score. When there was not agreement, a consensus score was adopted.

The loss by technical failure or inconclusive results has made it impossible to evaluate the immunohistochemical study of some samples, and these events were the reasons why they were excluded from the study.

The quantitative results were described as the mean and standard deviation. Qualitative data were described as frequencies. The correlation between the scores of the immunohistochemical expression of the proteins with the clinicopathological parameters was calculated by Spearman’s correlation coefficient. The Student’s *t*, χ^2^, Mann-Whitney and McNemar tests were used to assess the significance of the differences in the clinical and pathological parameters, and associations with proteins’ immunoexpression. The univariate logistic regression analysis (Analysis of Variance − ANOVA) and multivariate analysis were used to identify the dependent and independent variables. The statistical significance level was set at 5% (p<0.05), and the data were analyzed using the Statistical Package for Social Sciences^®^ software (SPSS^®^, Chicago, IL, USA), version 17.0.

The present study was conducted according to the ethical principles of the Declaration of Helsinki from the World Medical Association and has been approved by the Research Ethics Committees of our institution, CAAE: 0042.0.338.000-09.

## RESULTS

In terms of CRC, the average tumor size was 5.2±1.3cm (0.5 to 12cm). The size of the tumor along its longest axis was >5cm in 43 (67.1%) patients and ≤5cm in 21 (32.8%). Lymph node metastasis was found in 31 (48.4%) patients. The invasion of blood vessels was observed in 21 (32.8%) patients, lymphatic vessel invasion in 24 (37.5%) and neural invasion in 12 (18.7%). Eleven (17.2%) carcinomas were well differentiated; 51 (79.7%), moderately differentiated; and 2 (3.1%), poorly differentiated. The CCR superficially infiltrated (T1 + T2) the intestinal wall in 18 (28.1%) patients and deeply (T3 + T4) in 46 (71.9%). The presence of synchronous metastases was observed in 14 (21.9%) patients. Relapse occurred in 13 (20.3%) patients and 12 (18.7%) of them died for this reason. The average follow-up time was 19.1±2.8 months (3 to 36 months).

The average size of the polyps was 0.6±0.2cm (0.4 to 1.7cm). The polyps were <1.0cm in 34 (64.1%) patients and ≥1.0cm in 19 (35.8%). The histological type of adenoma was tubular in 49 (69%) and tubular-villous in 22 (31%). Moderate atypia were found in 39 (55%) adenomas and mild atypia was noted in 32 (45%).

The expression of beta-catenin protein in CRC was significantly (p<0.001) higher than in the adenomas and significantly (p<0.001) higher than in the adjacent non-neoplastic mucosa. This increased beta-catenin expression occurred mainly in the cytoplasm of cells, but nuclear staining was also significant. In adenomas, the staining of beta-catenin was significantly (p<0.0001) lower than in adjacent non-neoplastic mucosa (Table 1).

**Table 1:** Immunohistochemical staining of beta-catenin, adenomatous polyposis coli glycogen synthase kinase 3β (GSK-3β), axin 1 and ubiquitin in colorectal carcinoma, adjacent non-neoplastic mucosa, and colonic adenoma

Protein	Grade of immunohistochemical stained	CRC	Adjacent non-neoplastic mucosa	Adenoma	p value
		n (%)	n (%)	n (%)	
Beta-catenin		50 (78.1)	44 (68.7)	53 (74.6)	<0.001* (tumor *versus* adenoma)
	Strong	37 (74.0)	22 (50.0)	8 (15.1)	<0.001* (tumor *versus* mucosa) <0.0001* (adenoma *versus* mucosa)
	Reduced	13 (26.0)	22 (50.0)	45 (84.9)	
APC		42 (65.6)	42 (65.6)	53 (74.6)	<0.0001* (tumor *versus* adenoma) 0.24, NS (mucosa *versus* tumor) <0.0001* (adenoma *versus* mucosa)
	Strong	38 (90.4)	41 (97.6)	24 (45.2)	
	Reduced	4 (9.5)	1 (2.4)	29 (54.7)	
GSK3β		54 (84.4)	54 (84.4)	46 (70.4)	0.0005* (tumor *versus* adenoma) 1.00, NS (tumor *versus* adenoma) 0.0005* (adenoma *versus* mucosa)
	Strong	46 (85.2)	46 (85.2)	6 (13.1)	
	Reduced	8 (14.8)	8 (14.8)	40 (86.9)	
Axin 1		61 (95.3)	49 (76.5)		0.04*
	Strong	34 (55.7)	13 (26.5)		
	Reduced	27 (44.3)	36 (73.4)		
Ubiquitin		48 (75.0)	48 (75.0)		0.3, NS
	Strong	18 (37.5)	29 (60.4)		
	Reduced	30 (62.5)	19 (39.6)		

* Significant. CRC: colorectal carcinoma; NS: non significant; APC: adenomatous polyposis coli.

The immunoreactivity of the APC protein in CRC was significantly (p<0.0001) higher than in the adenoma. The APC protein immunoreactivity in the adjacent non-neoplastic mucosa was significantly (p<0.0001) higher than in the adenoma. There was no significant difference (p=0.24) between the immunoreactivity of the APC protein in CRC and in its adjacent non-neoplastic mucosa.

The immunoreactivity of the GSK3β protein in adenoma was significantly lower in the CRC and in the adjacent non-neoplastic mucosa (p=0.03 and p=0.0005, respectively). There was no significant difference (p=1.00) between the immunoreactivity of GSK3β protein in CRC and in the adjacent non-neoplastic mucosa.

The immunoreactivity of axin 1 protein was significantly (p=0.039) lower in the adjacent non-neoplastic mucosa. The expression of ubiquitin showed a significant difference (p=0.03) between the CRC and the adjacent non-neoplastic mucosa.

The immunohistochemical expression of beta-catenin protein APC, GSK3β, axin 1 and ubiquitin in colorectal adenomas and CRC did not show significant differences with the clinical and pathological characteristics of CRC and colonic adenoma.

## DISCUSSION

In humans, the beta-catenin protein is encoded by the gene CTNNB1, which maps at 3p22.1 (3,5). Hao et al.^(6)^ demonstrated an aberrant expression of beta-catenin mutations in the CCR, and this is an early event in human colorectal carcinogenesis. These authors verified that the adjacent normal epithelium to aberrant crypts showed a strong staining of beta-catenin, an event that was also observed in this study in CRC and in its adjacent non-neoplastic mucosa, but not in colonic adenomas.

The APC gene maps at position 21q in chromosome 5 and encodes a protein with multiple functional domains that interact with proliferation and apoptosis regulators.^(11,12)^ The gene is mutated in 63% of sporadic adenomas and in over than 80% of sporadic CRC. The heterozygous mutation is inherent in all cases of familial adenomatous polyposis.^(13-15)^ The APC protein forms molecular complexes that are capable of eliminating the intra-cytoplasmic beta-catenin, inducing its degradation.^(1,4,12)^ In the epithelium of the large intestine, APC expression is restricted to regions in which cell replication has ceased, and terminal differentiation was established.^(13)^

In the large intestine, isolated mutations in the APC gene are sufficient to provide a selective growth advantage, by reducing the function of the APC-specific degree sufficient to allow the accumulation of nuclear beta-catenin, promoting the cell proliferation without causing excessive apoptosis.^(2,3,12)^ Thus, since the physiological role of the APC protein is a growth advantage, the loss of its function will promote a cellular clonal expansion.^(11,12)^ When the APC protein is mutated, it loses its binding site in the complex destruction of beta-catenin, which causes an increased expression of beta-catenin protein in the cytoplasm and nucleus.^(15-17)^

Li et al.^(18)^ examined the expression of APC and mutated in CRC proteins, the key regulators of beta-catenin, by immunohistochemistry in right-sided serrated polyps. This study implied that the molecular-specific form of beta-catenin may participate in the Wnt-signaling activation of right-sided serrated polyps. Moreover, the loss of mutated in colorectal protein but not APC expression may contribute to the early activation of Wnt signaling in right-sided serrated polyps. In the present study, a decreased immunoreactivity of the non-mutated APC protein was observed in most patients with colonic adenomas. On the other hand, the significant presence of non-mutated APC protein in CRC and in adjacent non-neoplastic mucosa may indicate that the mutated APC protein is not involved as an important component in the process of carcinogenesis in these specific tumors. Wong et al.^(19)^studied 758 cases of colorectal adenomas and concluded that the malignant conversion of adenomas may not be related to the mutation of the APC gene alone. Thus, the development of colorectal carcinogenesis, even in the absence of the APC-mutated protein, is possible, as the results of the present study also suggested.

The GSK3β gene maps in chromosome 19 at position 13q.2. The GSK3β is a serine/threonine multifunctional protein, which is able to phosphorylate and inactivate glycogen synthase.^(20)^ The GSK3β protein acts as an inhibitory key toward the canonical-Wnt signaling pathway.^(21)^ The expression of the GSK3β gene attenuates the proliferation of CRC cells or leads to early apoptosis.^(22)^ Therefore, under physiological conditions, the GSK3β phosphorylates and degrades protein transcription factors and oncoproteins, which suggests that this enzyme would be a suppressive factor in tumor development, which negatively interferes with oncogenic signaling.^(21,23)^ It was also observed in the present study that the immunoreactivity of the GSK3β-mutated protein was shown to be increased in the CRC and in adjacent non-neoplastic mucosa regarding colonic adenoma.

Axin 1 has emerged as a major scaffold protein for regulating a variety of signaling pathways and biological functions.^(24)^ The human homolog gene of axin 1 was mapped to chromosome 16p13.3.^(25)^ In sporadic CRC, an increased expression of axin protein suggests that mutation of the corresponding gene can participate in colorectal carcinogenesis.^(26,27)^ In this way, the present study observed that the staining of the mutated protein GSK3β in CRC was significantly increased with respect to the non-neoplastic adjacent mucosa.

The ubiquitin (E3 ubiquitin ligase) is a protein that recruits an E2 ubiquitin-conjugating enzyme, which recognizes the protein substrate and assists or directly catalyzes the transfer of ubiquitin from the E2 ubiquitin-conjugating to the protein substrate.^(28)^ The human homolog gene was mapped to chromosome 17p16.38.^(28)^ In normal tissues, the phosphorylated beta-catenin is recognized by the E3 ubiquitin ligase complex forming the phosphorylated poly-ubiquitin/beta-catenin which is proteolyzed via the proteasome.^(29,30)^

Chen et al.^(7)^ found that the expression of ubiquitin in the CCR was significantly higher than in the adjacent non-neoplastic mucosa, and this result was also found in the present study. Moreover, they observed no significant relation between the expression of ubiquitin protein and the clinical and pathological features of CRC, a finding that was confirmed in this present study.

## CONCLUSION

The beta-catenin protein expression was increased in the cytoplasm and nucleus of the neoplastic cells, when compared to the colonic adenoma. The adenomatous polyposis coli protein was mainly altered in adenomas, and that, along with the increase in colorectal cancer of GSK3β, axin 1 and ubiquitin-mutated proteins, may prevent the phosphorylation of beta-catenin by the destroyer complex and its subsequent degradation in the proteasome. This event allows the stabilized beta-catenin protein to translocate into the cell nucleus, in which this protein can activate transcription factors, as well as the expression and activation of target genes related to colorectal carcinogenesis. Additional research is needed to determine whether the ongoing activity of signaling pathways is required for normal and neoplastic tissues, and whether or not these conditions differ sufficiently to allow for therapeutic intervention.
